# Reading Direct-Part Marking Data Matrix Code in the Context of Polymer-Based Additive Manufacturing

**DOI:** 10.3390/s23031619

**Published:** 2023-02-02

**Authors:** Daniel Matuszczyk, Frank Weichert

**Affiliations:** Department of Computer Science VII, Technical University Dortmund, 44227 Dortmund, Germany

**Keywords:** neural networks, additive manufacturing, direct-part marking

## Abstract

A novel approach to detect and decode direct-part-marked, low-contrast data matrix codes on polymer-based selective laser sintering manufactured parts, which is able to work on lightweight devices, is presented. Direct-part marking is a concept for labeling parts directly, which can be carried out during the additive manufacturing’s design process. Because of low contrast in polymer-based selective laser sintering manufactured parts, it is a challenging task to detect and read codes on unicolored parts. To achieve this, at first, codes are located using a deep-learning-based approach. Afterwards, the calculated regions of interest are passed into an image encoding network in order to compute readable standard data matrix codes. To enhance the training process, rendered images, improved with a generative adversarial network, are used. This process fulfills the traceability task in assembly line production and is suitable for running on mobile devices such as smartphones or cheap sensors placed in the assembly line. The results show that codes can be localized with 97.38% mean average precision, and a readability of 89.36% is achieved.

## 1. Introduction

**Figure 1:**
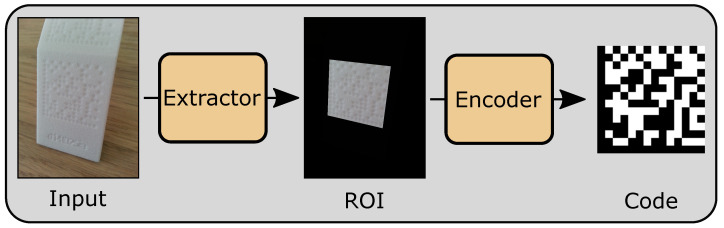
On the left side, a direct-part marking on an SLS part is presented. The same code is shown on the right side as standard data matrix code after passing the presented deep-learning-based processing pipeline.

Additive manufacturing (AM) is becoming more and more present in industrial environments, shifting from rapid prototyping and individual production to assembly line production systems to fulfill mass production tasks [1]. Industrial fields such as the automotive industries are interested in the advantages of AM design options keeping operation costs and processing times low [2]. A core component of automated assembly production lines is the traceability of manufactured parts. Tracking parts enables the identification of various problems in the production line, monitoring the manufacturing process and continuously improving quality requirements. Direct-part marking (DPM) is a concept to fulfill part traceability, where parts are labeled directly [3,4]. One advantage of DPM is the substitution of other marking approaches, such as radio frequency identification (RFID) chips or stickers, which saves necessary sensors. As methods for detecting DPMs in the form of data matrix codes on parts, which are manufactured with selective laser sintering (SLS), are missing a novel end-to-end approach based on deep learning (DL) to first localize the codes and afterwards read the codes is presented. In Figure 1, the difference between DPM and standard data matrix code is depicted. Perforated and planar surfaces are used to generate a data matrix code-like structure. The lighting conditions are important for contrasts on the surface. An image of a DPM part is passed into the extractor (Section 3.2) and is converted into standard data matrix code by the encoder network (Section 3.3).

The main focus is to provide an approach which is able to detect DPMs on AM-manufactured parts with sensors keeping hardware costs low and fulfilling part traceability requirements during each processing step. The applicability is demonstrated using AM-manufactured parts, as they can be perfectly used for DPMs, because DPMs are directly build on the part itself [5]. Manufacturing parts with integrated DPMs allows to gather information about the positioning during the build process and to identify possible placement parameter issues. This is a challenge for other marking techniques, as they can only be attached after the AM build process. By using deep learning methods for the extraction and data matrix encoding, the approach is able to overcome the challenge of missing contrasts of DPMs on SLS parts. Thereby, the approach has the capability of tracking manufactured parts, with common camera sensors on smartphones substituting other marking techniques and sensors. One challenge for deploying deep learning methods in industrial environments is to gather a huge amount of training data in order to ensure powerful models. The production of test parts will not only be expensive but also time-consuming, as assembly lines will be blocked for testing part creation. Therefore, the data limitation has to be considered for the DL part tracking approach.

Part traceability in manufacturing processes plays a crucial role in assembly line production. Part tracking ensures that information can be attached to its digital twin by capturing the specific tracking techniques [6]. This allows for gathering information in logs for specific items, generating a digital twin of the processed part. Through this process, storing batch numbers, serial numbers, timings and other useful information becomes easier. Knowing when a part passed different stages of production is indispensable for quality control. With part histories and online monitoring, weaknesses in the product chain can be detected. Even the costs of product recalls can be reduced, because it is possible to identify specific product batches with product tracking. Thereby, in case of quality issues, traceability can avoid recalling good parts with the knowledge of bad batches.

For part traceability, objects can be labeled with QR code, bar codes or data matrix codes [7]. These codes can be read automatically or by humans with scanners or cameras. It is also possible to track parts by using RFID chips attached to products [8]. Furthermore, parts can be tracked by observing their geometry aspects, but this technique is limited to only identify objects with different shapes. Depending on the labeling technique, different sensors are needed in order to track parts.

While stickers and RFID chips need to be attached to parts, the technique of DPM enables to label objects directly [3,4,5]. A direct labeling is called direct-part marking, where, e.g., metal parts can be manipulated with lasers to attach data matrix codes onto part surfaces [9]. Sticking to the data matrix ISO standard (ISO/IEC 16022:2006), the typical black–white mark can be transformed to perforated and planar regions. Using AM with selective laser sintering enables the possibility of building parts with labels by attaching the codes to a part during the design process.

Selective laser sintering is an AM technology, where a part is printed based on a given input model [10]. As the input model can be manipulated directly, the DPMs can be attached before the building process. During the SLS process, a thermal source is used to sinter powder particles in a powder bed based on the input model. Often, a white polymer is used, resulting in parts with low contrasts, which is a huge challenge, as common code detection techniques often rely on edge detection [11,12]. A white material will lead to a white data matrix code but, instead of having a black and white code, the code consists of planar surfaces and hollows. In Figure 1, a data matrix code on a SLS part is presented, where the code consists of planar surfaces and hollows instead of standard black and white code. By detecting the perforated and planar surfaces, it is possible to read the data matrix code. To read a code, the alignment, light conditions and camera position are important, as the material is completely white, and contrast is only given by the hollow parts.

In order to track parts, at first, a localization of part labels has to be calculated and, afterwards, the label information needs to be read. This leads to the well-known problem statement of object detection in computer vision,. In computer vision class prediction and object localization is achieved due to various algorithms (see Section 2). As the labels are created directly on the part, the color of the data matrix code will be one color and the same as the part color. Detecting these marks with an absence of contrast is where existing strategies fail. This yields an approach capable of detecting these specific marks.

The main contribution of this paper is the combination of a deep-learning-based network for localization with a convolutional neural network encoder. Using this combination, single-color low-contrast codes on parts can be transformed into standardized data matrix codes. These codes can then be read by a standard data matrix library. This procedure enables an end-to-end trainable approach. The end-to-end learning approach ensures an adaptive and generic processing pipeline without the need for different parameter settings. By keeping the model size lean, the architecture is small enough to run on a mobile device, making it possible to mount sensors on the assembly line or to check labels by humans with just a mobile phone during certain stages, which reduces the amount of needed sensors. This extends the design space for part tracking solutions as data matrix codes, which can be attached during the building process, in contrast to other solutions (RFID and stickers) [13].

The paper’s structure is as follows. After the introduction, related work is presented in Section 2. The main contribution for detecting and reading data matrix codes is described in Section 3. Afterwards, aspects of the dataset generation and the results are presented in Section 4. In Section 5, the results are inspected, and finally, a brief conclusion is drawn in Section 6.

## 2. Related Work

The Section 2 addresses techniques for DPM and possibilities to find and read code patterns in unstructured environments, followed by a motivation for the need of a novel approach to detect DPMs on SLS-manufactured parts. A brief introduction about AM processes and their relation to other manufacturing processes in context of DPMs is given. For DPMs, the main focus of research lies in the conception and production of DPMs mostly using established applications to read codes, while in this approach, the reading process is focused [3,14]. Therefore, image processing techniques are addressed, and especially, DL methods and their performance under data limitation are highlighted.

One advantage of AM in the context of DPM is the possibility to directly build the DPM during the printing process. AM technologies can be grouped into different process categories based on different technologies with varying materials, such as powder bed fusion, binder jetting or material extrusion [15]. The different process categories and materials are crucial for the characteristics of DPMs on parts. As an example, DPMs on parts manufactured with material extrusion techniques, such as fused deposition modeling (FDM), are connected often [16]. Furthermore, often, non-powder-bed fusion manufactured parts, such as material extrusion, are mostly built one after the other. Therefore, the traceability aspect with DPMs for production lines is of less importance, as other labeling techniques can be applied. In addition to AM technologies, DPMs can also be attached to parts after the build process, using techniques such as drilling or laser direct marking [17,18]. These marking techniques require an additional processing step after the part building, resulting in an information loss of the part position in the powder bed. Additionally, these techniques produce markings with more contrasts, which can also be read by more traditional edge detection methods.

Traditional approaches for reading markings such as bar codes, QR codes and data matrix codes often use computer vision edge detection methods. Methods such as blob detection, canny edge detection and Hough transformation are used to detect drilled holes in wood-based materials [19]. A canny edge detector based algorithm is also used to detect holes on printed circuit boards [20]. Belonging to other domains, these approaches can be transferred to the detection of codes. Huang et al. used an edge-based detection with LSD and RANSAC algorithms to detect codes, and Gaur et al. used grayscale conversion and edge detection followed by morphological operations to search connected components and segment bar codes [11,12]. Another edge-based approach is used by Kulshreshtha et al. to perform robustness analyses of QR and data matrix codes using grayscale conversion with binary conversion and edge detection [21]. These approaches are presented for traditional markings, not for DPMs, and rely on the high contrast of the black/white code structure. Karrach et al. propose a method to detect data matrix codes for production engineering [7]. They find regions of interest by connected components labeling on binary images filtered with minimal area size conditions. The method is validated on images of data matrix codes on metal parts attached directly by laser technique. Although the proposed method tackles the direct-part marking problem for part manufacturing, laser-marked metal parts show high contrast between black and white data matrix components. Binary representations of images taken from SLS-manufactured parts with data matrix codes show a lot of noise due to the lack of contrast. Because of this, a more flexible approach to find and detect data matrix codes is needed. In the context of sand casting, Uyan et al. present how direct-part markings can be read with tablets or mobile phones by free bar code readers [14]. The focus is on the production and analysis of DPMs in the form of data matrix codes and underlines the need for high contrasts for readable tags but, as common bar code readers fail to read DPM on SLS parts, this underlines the need for a novel approach.

Deep learning (DL) approaches are more adaptive in contrast to traditional approaches. This is why DL is common to perform object detection, resulting in common models such as YOLO, Faster R-CNN and Mask R-CNN, differing in speed and accuracy [22,23,24]. Several approaches for code detection with DL exist already. Zharkov et al. propose a network for detecting universal codes such as QR codes, data matrix codes and bar codes [25]. They use a two-stage pipeline by running a semantic segmentation to find relevant regions and then classifying the detected bar code. A YOLO-based approach is used by Xiao et al. to achieve bar code detection for supermarket product labels [26]. Moreover, the Faster R-CNN architecture can be used to perform code detection [27,28,29]. Another method for detecting data matrix codes is presented by Li et al., where multiscale prediction with a convolutional neural network is used to detect multiscale data matrix codes [4]. These DL-based approaches need to be trained with appropriate training material. The availability of training data for DL models in industrial environments is limited. An approach to limit gathering real-world data is the use of synthetic rendered images for training steps and data augmentation. However, even with domain-specific renderers, there is still a reality gap to close [30]. As an example, a machine learning dataset for metal AM can be enriched by applying image augmentation techniques such as GANs on given images [31]. As a general approach, a ConSinGAN for data augmentation on existing training images may be used [32].

Combining the results of the mentioned methods, DL for code detection is a reasonable approach. In contrast to the proposed methods with detection designed for standardized codes with the typical black/white format, the SLS-manufactured parts have a completely different appearance. As the approaches mainly focus on detecting codes in environments where parts have more contrast, as in SLS manufacturing, a different approach for detecting and interpreting low-contrast unicolored data matrix codes is needed. Therefore, a localization strategy such as a Faster R-CNN architecture seems to be a reasonable approach. Furthermore, an augmentation strategy in order to enhance the training process is a valid option. By establishing a module to transfer extracted DPMs to data matrix codes, the final step for reading codes is taken.

## 3. Materials and Methods

**Figure 2:**
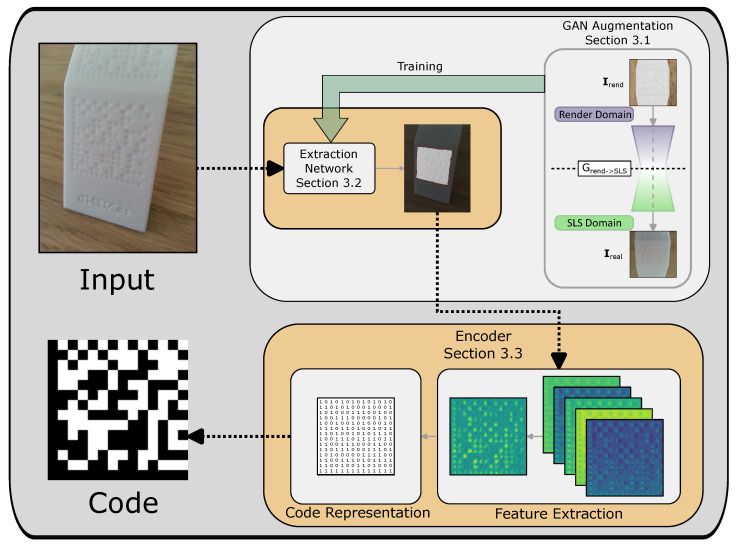
The approach’s steps for reading a data matrix code are presented. DPM areas are extracted (Section 3.2) and transformed into standard data matrix code (Section 3.3).

The presented approach is based on deep learning models for extracting and reading data matrix codes on SLS-printed parts. The method can be used to scan parts manually with a mobile phone or by placing cameras on top of processing lines. Therefore, the method considers pictures of parts placed close to the camera and aims to achieve low memory consumption. As depicted in Figure 2, an input image is passed to an extractor (Section 3.2) computing a segmentation for region of interests (ROIs) with an accurate masking. Afterwards, the extracted region is passed to an encoder (Section 3.3) in order to produce standard data matrix code. Furthermore, to enhance the extractor’s training process, a generative adversarial network (GAN) is used to generate and augment training data to overcome data limitations in production pipelines (Section 3.1) [33].

The first step is to render synthetic images refined by a GAN in order to reduce the process of gathering enough real-world images, which is a time-consuming task in industrial environments. Therefore, training the models becomes more accessible, even with limited data availability [33]. To extract exact pixel-based regions of the data matrix codes, an extractor based on a region proposal network (RPN) with binary mask predictions for each region of interest is deployed [22]. This results in binary image mask predictions, which are combined with a four-point transformation of the minimal area enclosing the mask to extract images only containing the data matrix code. The minimal containing rectangle is computed by calculating the convex hull of the mask prediction and a rotating calipers approach to fit the rectangle [34]. Afterwards, an encoder model (Section 3.3) is designed to transform low-contrast direct-part markings into readable standardized black/white data matrix codes. For this, the data matrix extractions are parsed as input to the encoder network, and a matrix with binary values is computed to represent the data matrix code. This opens the opportunity to scan DPMs by humans with mobile phones or to place cheap cameras above assembly lines.

### 3.1. GAN Image Augmentation

To overcome the data limitation issue, a generative adversarial network (GAN)-based architecture for realistic SLS photo creation after rendering synthetic images is introduced [35]. With a cycle GAN model, the image’s specific characteristics are learned and can be applied as a transformation to enhance the realistic appearance of rendered images [33].

To create synthetic images, an approach for rendering photographs of 3D models is deployed. Therefore, different lighting conditions and camera positions combined with a bidirectional scattering distribution function (BSDF) with GGX are used to create a rendered image $I_{rend}\in {\left[-1,1\right]}^{C\times H\times W}$, with C, H and W representing color channels, height and width and color values being normalized in the interval [−1,1] [36]. For the lighting conditions and camera positions, the same angles as in the real-world dataset (see Section 4) are used.

Using a cycle GAN approach, a domain shift between rendered and real images of SLS parts becomes possible. Furthermore, due to the cycle GAN approach, an unpaired image training is possible, which has the advantage that rendered and real SLS images can have some misalignment. The model’s generator is trained to learn a transformation $G_{rend\to SLS}:I_{rend}\to I_{SLS}$ and converts each rendered image $I_{rend}$ which is passed to it into a fake image $I_{SLS^{\prime }}$ containing information of the real SLS domain [33]. For the cycle consistency, a generator $G_{SLS\to rend}$ is used to perform the opposite task. Furthermore, discriminators $D_{SLS}$ and $D_{rend}$ are used to distinguish between the two domains, e.g., $D_{SLS}$ is trained to distinguish between a real image $I_{SLS}$ and images generated by the generator $G_{rend\to real}$ [33]. The architecture for the generators consists of three convolutions, nine residual blocks and a 70×70 PatchGan for the discriminators [33]. The loss is calculated by combining generator loss, discriminator loss and the cycle consistency loss [33].

**Figure 3:**
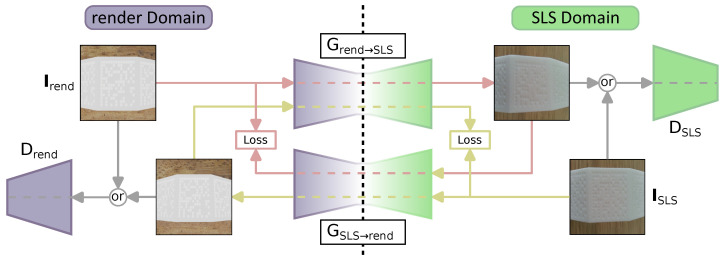
Overview of the GAN-based image augmentation. Images are transformed from the rendered domain to the real-world domain. The cycles are used for loss calculation and marked as red (rendered to real) and light yellow (real to rendered).

As depicted in Figure 3, the rendered image’s ($I_{rend}$) characteristics are transferred to the real-world SLS domain in order to enhance the image’s appearance close to real-world pictures ($I_{SLS}$). Therefore, rendered images are passed into the generator acting as a function to transform rendered into real-world images. Utilizing the discriminator, the images are scored as real or fake. The loss is calculated by combining generator, discriminator and cycle loss. In Section 4, it is shown that the images not only look similar to human eyes but also improve the deep learning model’s training process.

By first rendering images from 3D models of the parts and afterwards refining the rendered images, the specific characteristics of real images are captured. This enables the use of synthetically created images for the data matrix code extraction model (see Section 3.2) to improve the effectiveness under training data limitations, such as in real-world industry assembly lines.

### 3.2. Data Matrix Extraction

In industrial environments, code detection is possible in certain ways, e.g., scanning codes with mobile phones or installing scanners above production lines. Finding the data matrix code is an important step to preprocess given input images in order to read the codes. The extraction should be both lightweight and able to detect multiple codes at once. Therefore, an approach is deployed to find code regions and extract each region as a single image.

Before code areas can be passed into the encoder network, the code has to be extracted from certain images. For this purpose, images are processed with the usage of deep-learning-based mask segmentation with a ResNet-50-FPN backbone [22,37]. The model’s backbone is pretrained on the *ImageNet* dataset to obtain initial model weights [38]. Faster R-CNN is already used for real time data matrix code detection. For this approach, adding the overhead of mask segmentations for more accurate results is necessary for preprocessing exact bounding boxes instead of axis-aligned bounding boxes [22,27].

At first, possible object candidates are identified with the model’s region proposal network (RPN), and then binary masks, class scores and bounding boxes are computed. For a given input image $I_{in}\in R^{W\times H\times C}$ with width, height and color W,H,C∈N, this leads to images $I_{in\_mask\_x}\in {\left[0,1\right]}^{H\times W}$ and x∈N being instances of codes in the input image $I_{in}$. For model training, the synthetic images from Section 3.1 are used to reduce the amount of real training images. After obtaining the binary mask $I_{in\_mask}$ for each data matrix code region of $I_{in}$, a minimal rotated rectangle for each mask leads to four corner points of the rectangle [34]. The four corner points can then be used for the localization of the data matrix code area.

### 3.3. Data Matrix Encoding

**Figure 4:**
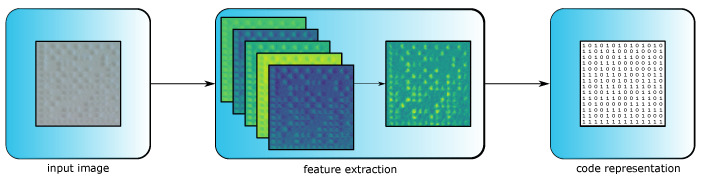
An input image is processed by the encoder model. First, convolutional layers followed by normalization are used to extract multiple features. Here the features are presented as abstract feature maps. The feature maps are aggregated by a last convolutional layer and afterwards, a matrix representation for the code is computed by a fully connected layer combined with a sigmoid function.

In order to read DPM on SLS parts, a novel approach that is more flexible to lighting conditions and low-contrast surfaces is needed. Therefore, a small and lightweight deep learning approach to encode DPM on SLS parts into matrices representing data matrix codes is presented. The process of the approach is depicted in Figure 4, where an input image is converted to a matrix representation after running through several encoding steps.

After the data matrix extraction (see Section 3.2), the images containing the code need to be transformed into readable data matrix code. Therefore, the goal of the encoder structure is to compute a matrix $B_{code}\in {\left[0,1\right]}^{W\times H}$, where W,H∈N is the size of the data matrix code. Given an input image $I_{DPM}\in N^{W^{\prime }\times H^{\prime }\times C}$ with C=3 as color information of an RGB image of width and height $W^{\prime },H^{\prime }\in N$, the model is able to learn a mapping $I_{DPM}\to B_{code}$. The encoder consists of four convolutional layers combined with instance normalization layers, followed by a fully connected layer to extract features and afterwards classify notches and planar surfaces [39].

For an input image $I_{DPM}$, the features are computed stepwise. A layer is presented by the tensor $X_{l}^{W_{l}\times H_{l}\times C_{l}}$, with l∈N indexing specific layers and $X_{0}=I_{DPM}$ representing the input. The first convolutional layer computes 32 features, the second 64 and the third 128. All three layers use a kernel size of 3×3 with stride and padding set to 1. For an input image with a width and height of 180, the image dimension remains 180×180. The fourth convolutional layer compresses the extracted feature, resulting in the matrix $X_{4}\in R^{180\times 180}$ by using a 1×1 kernel with stride set to one. After performing feature extraction on the image, a fully connected layer maps the extracted features of $X_{4}$ into $X_{5}\in R^{14\times 14}$ which, after applying a sigmoid function, leads to the matrix $B_{code}\in {\left[0,1\right]}^{14\times 14}$. $B_{code}$ is compared against an annotated matrix $B_{annotation}\in {\left[0,1\right]}^{14\times 14}$, and thereby, the encoder is able to learn the mapping $I_{DPM}\to B_{code}$.

To perform error handling for miscalculations in the finder pattern, the correct pattern can be predicted calculating the two edges with the matrix entries which contain the most entries scored as black as the finder pattern of the data matrix code, directly leading to correct patterns. To obtain a standard data matrix code, the zeros and ones of $B_{code}$ are converted into black and white squares. The resulting standard data matrix code is now able to be read by common code readers.

As the model only consists of few layers, the number of parameters is still low enough to run on a lightweight device. Taking advantage of deep learning’s flexibility, the approach can output data matrix codes even with mobile phones under different lighting conditions for DPMs on low-contrast SLS-manufactured parts.

## 4. Results

To depict the performance of the proposed pipeline for detecting low-contrast data matrix codes on SLS-manufactured parts, first the results of the code extraction (see Section 3.2) step from images are measured. Afterwards, the results of the encoding architecture (see Section 3.3) are shown. For both models, an 80% train and 20% test setup is used, with the results being computed on the 20% test dataset. To achieve more meaningful results, five random splits on the datasets are created. For implementation and the experiments, Pytorch 1.7 is used [40].

As there are no available datasets for detecting data matrix codes on SLS-manufactured parts, a dataset is created representing the desired conditions. The dataset for localization consists of 204 images containing 299 data matrix codes. The data matrix codes are represented by hollows and planar sequences varying in diameter and depth. For the encoder model, 299 data matrix code images are used. The images are taken with the following camera: sensor type: CMOS BSI; sensor size: 4.6×3.45 mm; pixel size: ≈1.409μm; and resolution: 3264×2448. Each image varies in distance from three to ten centimeters to the focused data matrix code. Furthermore, images are taken with a camera position relative to the data matrix codes at 0 degrees, representing direct focus and ranging from −30 degrees to 30 degrees. Moreover, the lighting conditions vary, as direct light is rotated around the objects and the images are taken with either direct light or ambient light. Examples of the images are presented in Figure 1, Figure 2 and Figure 3. In addition to the results, the approach was also deployed as an android app to ensure the method’s applicability for production line environments in use cases where humans scan DPM on parts with mobile phones. Therefore, the functionality was tested on an Honor 6x (Octa-Core 4 × 2.10 GHz and 4 × 1.70 GHz CPU, 3.0 GB RAM). This ensures that the approach’s models are lean enough to run on a common smartphone.

For the code extraction evaluation, the COCO datasets metrics are used focusing on pixel-based segmentation [41]. The COCO metrics average over multiple intersections over union thresholds (IoU), where the threshold is taken from [0.5,0.95] in 0.05 steps. Normally, the COCO metrics are calculated for different object scales, but since the dataset only consists of large objects, only the large scale is considered. The metric uses average precision (AP) and average recall (AR) representing values to evaluate how accurate predictions are and how good the model performs on finding positive cases. For the experiments, the results are computed not only for the full dataset but also for combinations with synthetic images (see Section 3.1) and parts of the real dataset only. The results for the COCO metrics are presented in Table 1. The metrics are calculated for 25%, 50% and all of the 204 images for the code localization. Therefore, the impact of enriching parts of the dataset with the synthetic images becomes visible.

Results for the data matrix encoding model (see Section 3.3) are obtained by reading the 299 transformed black/white standardized data matrix codes with the Dynamsoft Barcode Reader (DBR) version 8.8.0.0831 (https://www.dynamsoft.com/, last accessed 28 November 2022). If the reader converts the data matrix code into the same string representation as labeled, the reading is classified as successful. As a score, the precision is used with TP being true positives and FP false positives, the precision is $\frac{TP}{TP+FP}$. The DBR also comes with a module for reading direct-part markings, which is compared with the provided solution by inputting extracted data matrix codes. The results for the different test splits on the dataset for data matrix code reading are presented in Table 2.

## 5. Discussion

As a concept is needed to read direct-part markings on SLS-manufactured parts to track them in assembly lines, a solution for localization and reading data matrix codes with DL is presented. Summarizing the results, the adaptive model is able to achieve the desired goal under given conditions. For data matrix localization, a mAP of 96.95% is reached, while the readability achieves an average of 87.07%. Furthermore, the synthetic images were used to improve the training process and limit the amount of needed data.

The localization of data matrix code is an important task for maintaining the ability of tracking parts in different environments. As most applications tackle the reading of standardized black/white codes, the presented approach is designed to read data matrix codes on low-contrast, completely white surfaces with planar or hollow cells, where the data matrix code is added during the design phase and printed directly with the part [25,27,28]. The trained architecture is able to compute binary masks for data matrix code locations with an mAP of 96.95% (see Table 1). By using synthetic images for the training process, it is depicted that under data limitation the GAN-finished rendered images improve the training process. This is useful to maintain production lines as the gathering of training data becomes cheaper. As there are no similar approaches, a comparison is not possible. The test images mostly contain only one large data matrix code, which is considered as a use case where a manufactured part is scanned close to the camera. These images support the localization process, and therefore the scores are quite high. Nevertheless, this is a possible use case, and these approaches are known to achieve good segmentation results, above all for specialized use cases. Transforming the image with the computed region results in a clipping which only contains the data matrix code and is now prepared for the reading step. This shows that the architecture is able to achieve an exact segmentation of codes which is needed for the next processing step.

While reading a standardized black/white data matrix code is a trivial step for existing libraries, the special images cut out by the localization architecture only contain an image with contours from light conditions, camera position and spatial components of the perforated and planar surfaces of the manufactured part, which is completely white. Although with the Dynamsoft Barcode Reader and its DPM module there is a reader for DPMs, the reader achieves quite low scores because it is not specialized in reading codes on SLS parts. This can be traced back due to the reader’s DPM module, which is designed for reading DPMs on metal parts which have been attached by laser technique. These DPMs result in high-contrast images because the laser burns black surfaces into the metal. Karrach et al. [7] compared the Dynamsoft Barcode Reader and several other readers for reading DPMs on metal parts, in which the DBR achieved better results. Thereby, a comparison is not really meaningful but underlines the need of generic architectures, especially a shift for readers to low-contrast domains to enable the use of DPM in SLS manufacturing.

With reading scores of 87.07%, there is still a gap to close but, considering that scanners can pass more photos, it is possible to achieve a good read after a reading failure. It should be pointed out here that data matrix code itself contains a redundancy supporting the reading process if the network miscalculated data matrix code entries.

The approach’s architecture allows a flexible way to perform annotation on new images. As annotations for localization are represented by masks and bounding boxes, it is only necessary to annotate the location of the data matrix code. Due to the rendering step and GAN model, it is possible to create synthetic data which limit the annotation process, because less data are necessary. The encoder module makes it possible to compare computed results against a binary matrix. This avoids an annotation process where each cell needs to be annotated. The memory consumption for reading one data matrix code image takes about 2GB and, therefore, is small enough to run on lightweight devices.

## 6. Conclusions

In this paper, an approach for reading data matrix codes on SLS-manufactured parts is presented, which is an important key component for part tracking in assembly lines in AM. These data matrix codes are special due to their spatial expression with hollows and planar surfaces on single-color materials. The approach for the localization and extraction of data matrix code areas on manufactured parts is based on a deep learning architecture. The localization network is able to extract the desired code regions in order to pass them to a new encoder model with an mAP of 97.38%. The training process can be enhanced by creating synthetic data with image rendering and GAN refinement. The encoder model is able to transform nonreadable low-contrast spatial DPM into standardized black/white data matrix code. The transformed codes can be read with an average of 85.99%, which is a good score to support part tracking for AM assembly lines. All in all, it can be assumed that the presented approach is a good starting point to ensure part traceability with DPM in the context of SLS additive manufacturing as being flexible and lightweight.

## Figures and Tables

**Table 1:** Presented here are the results for COCO metrics mean average precision (mAP) and mean average recall (mAR) for the five different test splits and as average values. The metrics are calculated for models trained with 25%, 50% and 100% of the 204 real images (e.g., 25% real). Furthermore, the real images are combined with the synthetically created images (e.g., 25% real + GAN).

Ratio Gan/Real	Metric	Average	Split1	Split3	Split3	Split4	Split5
25% real	mAP	77.02	89.20	71.12	83.17	76.69	39.15
25% real	mAR	77.71	91.03	73.79	83.10	76.21	45.86
25% real + GAN	mAP	85.67	89.72	85.93	83.15	87.93	78.38
25% real + GAN	mAR	86.43	91.38	84.83	85.17	89.31	77.59
50% real	mAP	92.09	95.48	94.10	89.11	89.32	92.84
50% real	mAR	93.33	96.21	94.48	90.69	86.201	94.83
50% real + GAN	mAP	92.23	96.25	91.96	87.10	93.58	92.25
50% real + GAN	mAR	93.53	96.63	93.45	88.62	94.83	94.14
100% real	mAP	96.62	97.40	97.36	96.39	96.30	95.65
100% real	mAR	97.20	97.59	97.93	97.24	96.90	96.34
100% real + GAN	mAP	96.21	96.28	96.35	93.86	96.80	97.78
100% real + GAN	mAR	97.38	96.90	97.59	95.52	98.28	98.62

**Table 2:** Presented here are the results for the encoder as precision compared with the Dynamsoft Barcode Reader for DPM on SLS-manufactured parts for the five different test splits and as average values. With an average of 85.99%, the encoder is able to transform images into readable data matrix code. As the DBR is not deployed for SLS DPM, an average of 18.08 indicates that a comparison is not fair.

	Average	Split1	Split3	Split3	Split4	Split5
encoder	85.99	89.36	88.30	86.67	81.91	83.70
dbr	18.08	18.09	23.40	14.44	14.89	19.57

## Data Availability

Not applicable.
